# Assessment of axial spondyloarthritis activity using a magnetic resonance imaging-based multi-region-of-interest fusion model

**DOI:** 10.1186/s13075-023-03193-6

**Published:** 2023-11-24

**Authors:** Peijin Xin, Qizheng Wang, Ruixin Yan, Yongye Chen, Yupeng Zhu, Enlong Zhang, Cui Ren, Ning Lang

**Affiliations:** Department of Radiology, Peking University Third Hospital, Beijing, People’s Republic of China

**Keywords:** Axial spondyloarthritis, Disease activity, Magnetic resonance imaging, Radiomics

## Abstract

**Background:**

Identifying axial spondyloarthritis (axSpA) activity early and accurately is essential for treating physicians to adjust treatment plans and guide clinical decisions promptly. The current literature is mostly focused on axSpA diagnosis, and there has been thus far, no study that reported the use of a radiomics approach for differentiating axSpA disease activity. In this study, the aim was to develop a radiomics model for differentiating active from non-active axSpA based on fat-suppressed (FS) T2-weighted (T2w) magnetic resonance imaging (MRI) of sacroiliac joints.

**Methods:**

This retrospective study included 109 patients diagnosed with non-active axSpA (*n* = 68) and active axSpA (*n* = 41); patients were divided into training and testing cohorts at a ratio of 8:2. Radiomics features were extracted from 3.0 T sacroiliac MRI using two different heterogeneous regions of interest (ROIs, Circle and Facet). Various methods were used to select relevant and robust features, and different classifiers were used to build Circle-based, Facet-based, and a fusion prediction model. Their performance was compared using various statistical parameters. *p* < 0.05 is considered statistically significant.

**Results:**

For both Circle- and Facet-based models, 2284 radiomics features were extracted. The combined fusion ROI model accurately differentiated between active and non-active axSpA, with high accuracy (0.90 vs.0.81), sensitivity (0.90 vs. 0.75), and specificity (0.90 vs. 0.85) in both training and testing cohorts.

**Conclusion:**

The multi-ROI fusion radiomics model developed in this study differentiated between active and non-active axSpA using sacroiliac FS T2w-MRI. The results suggest MRI-based radiomics of the SIJ can distinguish axSpA activity, which can improve the therapeutic result and patient prognosis. To our knowledge, this is the only study in the literature that used a radiomics approach to determine axSpA activity.

**Supplementary Information:**

The online version contains supplementary material available at 10.1186/s13075-023-03193-6.

## Background

Axial spondyloarthritis (axSpA) is a group of chronic autoimmune diseases characterized by inflammation in the spine and sacroiliac joints (SIJs) and peripheral arthritis in the lower limbs [1]. Disease activity refers to the severity of a disease at a given moment in time and is an important prognostic and therapeutic index in axSpA patients. In clinical studies, disease activity has been used to measure how well a treatment works to reduce symptoms and provides strong evidence for research. For obtaining the best possible health-related quality of life, the control of disease activity has a prominent place in the management of axSpA [2, 3]. Being able to measure disease activity more accurately can improve when and how treatment decisions are taken, therefore improving the outcome for patient management.

Traditionally, different scales have been used to measure and compare disease activity, such as Bath Ankylosing Spondylitis Disease Activity Index (BASDAI) [4] and Ankylosing Spondylitis Disease Activity Score (ASDAS) [5]. These scales are mostly based on patient-reported outcome measures, which are numerical or visual analog self-assessments of the patient’s symptom severity [6]. Additionally, ASDAS includes laboratory results such as erythrocyte sedimentation rate (ESR) or C-reactive protein (CRP) and is currently considered the gold standard for disease activity assessment in a clinical setting [7]. Being at least partially patient-driven, these two scales are inevitably influenced by many confounding subjective factors, including coexisting morbidities of the patients and different views that patients and physicians have on what disease activity means [8–10]. This results in significant variation when quantifying the levels of pain or discomfort and makes it difficult to compare assessments performed across different patients, institutions or consultations.

MRI is a sensitive imaging technique for early diagnosis of inflammatory sacroiliitis for axSpA patients with lower back pain owing to its high textural contrast resolution [11]. MRI reveals signs of inflammation, including active signs like bone marrow edema (BME) and chronic structural signs such as erosion [12]. Moreover, as an objective tool, MRI scans acquired under similar scanners and parameters have a high degree of comparability. Although different scales exist to quantify BME in SIJs of axSpA patients, they have many shortcomings which limit their use in clinical studies. Hence, an objective computerized method using SIJ-MRIs to determine axSpA disease activity has the potential of overcoming these limitations, serving as a complement or alternative in situations where patient-reported outcomes might be compromised.

In medical imaging, radiomics refers to high-throughput computational extraction of quantitative features from a certain region of interest (ROI) [13]. Radiomics translates medical digital images into deep-level data for quantitative analysis, which could ultimately aid diagnosis or classify or grade diseases [14]. Radiomics has become a focus in recent medical imaging research, especially in the field of oncology [15–18].

As a low-cost and noninvasive image processing method, radiomics has immense potential for axSpA evaluation. Owing to the complexity and heterogeneity of musculoskeletal medical imaging, the application of radiomics in diseases, including axSpA, is still in its initial stages. Previous studies have reported the use of radiomics methods to analyze SIJ-MRIs [19–21]. These studies suggest that a SIJ-MRI-based radiomics model can enhance the efficacy in differentiating axSpA, thereby facilitating the clinical decision-making process. However, current literature is mostly focused on axSpA diagnosis, and thus far, no study has reported the use of radiomics for differentiating axSpA disease activity. Moreover, previous studies focusing on various types of cancer have showed that a combined radiomics model derived from different ROI has high predictive value, which can improve clinical decision-making [22–24]. While previous studies evaluating axSpA only analyzed one type of ROI, no reports compared the performance of models combining or constructed using different ROIs.

In this study, we aimed to retrospectively investigate the ability of radiomics features extracted from two different types of ROI that are segmented from SIJ-MRI to differentiate disease activity in axSpA, as well as to establish a radiomics models for the differentiation of active and non-active axSpA.

## Methods

**Fig. 1:**
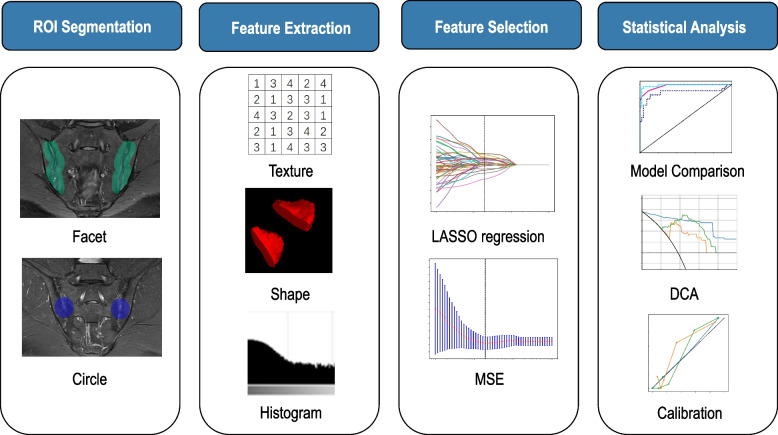
Workflow of the study. Manual segmentation and automatic expansions were performed on SIJ-MRIs to generate two ROIs, Circle and Facet. Different features were extracted, then selected by various methods, including cross-validation, LASSO, and MSE. Different predictive models were constructed using these features. Their performance was assessed using the ROC, decision, and calibration curves

This study was reviewed and approved by the Institutional Research Ethics Board, which adheres to the tenets prescribed by the Declaration of Helsinki (institutional review board M2022399, National Clinical Trial number MR-11–22-009236). Being retrospective research, the need for signed informed consent was waived. The workflow of the radiomics analysis conducted in this study is illustrated in Fig. 1.

### Dataset

Approval was obtained for the use of patient data. We identified 1096 consecutive patients who had undergone MRI for SIJs in our institution from April 2019 to September 2021. Inclusion criteria were as follows: (a) patients over 18 years old and (b) seeking medical attention for suspected axSpA, as diagnosed using the 2019 recommendations from the Assessment of SpondyloArthritis International Society (ASAS) working group.

Of the 1096 patients, 987 were excluded from this study for the following reasons: (a) absence of axSpA or lack of a definite clinical axSpA diagnosis, (b) sacroiliac MR scans having incomplete coverage or sequences, and (c) incomplete medical records, including a lack of CRP, ESR, BASDAI score, and ASDAS score.

Disease activity was assessed by two rheumatologists using the ASDAS calculated with CRP (ASDAS-CRP). An ASDAS-CRP score of greater or equal to 2.1 was considered high activity.

**Fig. 2:**
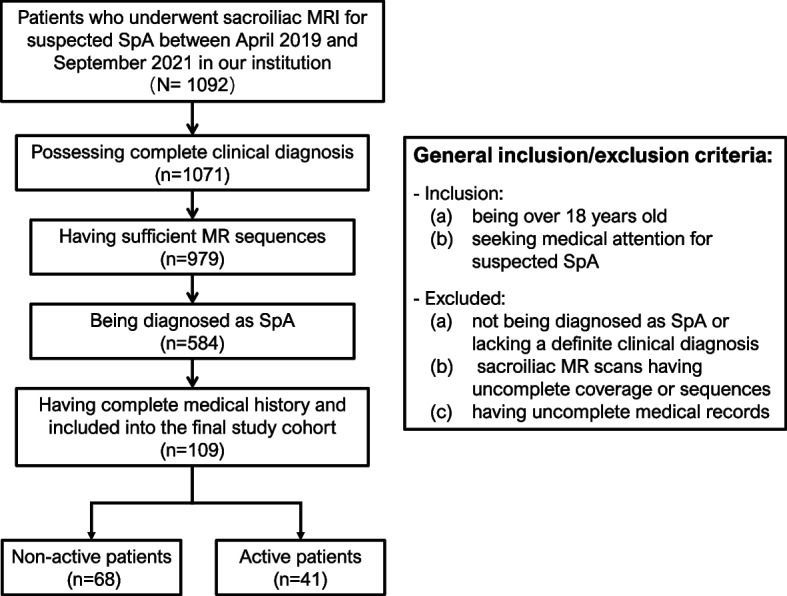
Flowchart for the inclusion of patients

The final cohort constituted 109 patients, of which 68 were non-active axSpA patients and 41 were active axSpA patients. The flowchart for the inclusion and exclusion of patients is shown in Fig. 2.

We randomly selected 80% of the samples as the training set, and the remaining 20% as the test set. Baseline characteristics of the final cohort can be found in Table 1.

**Table 1:** Baseline characteristics of patients with active and non-active axSpA in training and testing sets

	**Training set**	**Non-active patients**	**Active patients**	***P*** **value**	**Testing set**	**Non-active patients**	**Active patients**	***P*** **value**
Age (years)	31.68 ± 9.34	30.78 ± 7.05	33.15 ± 12.18	0.252	38.14 ± 10.56	35.57 ± 9.52	42.62 ± 11.40	0.135
CRP (mg/L)	2.37 ± 5.65	0.78 ± 0.71	4.98 ± 8.57	<0.001	1.83 ± 2.78	0.78 ± 0.74	3.65 ± 4.02	0.016
ESR (mm/h)	16.31 ± 18.04	7.69 ± 5.86	30.40 ± 22.08	<0.001	18.55 ± 23.77	9.21 ± 5.59	34.88 ± 34.03	0.011
BASDAI	3.04 ± 1.79	2.21 ± 1.05	4.40 ± 1.92	<0.001	3.39 ± 1.64	2.36 ± 0.87	5.19 ± 0.89	<0.001
ASDAS	1.93 ± 1.06	1.26 ± 0.45	3.04 ± 0.81	<0.001	1.83 ± 0.93	1.29 ± 0.47	2.78 ± 0.75	<0.001
Gender				0.964				0.234
Men	67(77.01)	41(75.93)	26(78.79)		20(90.91)	14(100.00)	6(75.00)	
Women	20(22.99)	13(24.07)	7(21.21)		2(9.09)	0(0.00)	2(25.00)	

*ASDAS* Ankylosing Spondylitis Disease Activity Score, *BASDAI* Bath Ankylosing Spondylitis Disease Activity Index, *CRP* C-reactive protein, *ESR* erythrocyte sedimentation rate

### Image acquisition

All MRI examinations were acquired using three 3.0-T scanners, two identical Discovery 750w (GE Healthcare, Milwaukee, WI) and one Discovery 750 (GE Healthcare, Milwaukee, WI). For both models of MR scanners, the same institutional protocol was used for SIJ examinations, including axial and oblique coronal (parallel with the long axis of the sacral bone), fat-suppressed (FS) T2-weighted (T2w) fast spin-echo (FSE) sequences as well as oblique coronal T1-weighted FSE sequences, and oblique coronal proton density-weighted FSE sequences.

We chose to acquire oblique coronal FS T2-weighted FSE images (scanning parameters: repetition time/time to echo = 3200/85 ms, slice thickness/gap = 4/0.5 mm, field of view = 30 × 30 cm, matrix size = 320 × 256, number of excitations = 4). This is because among the sequences included in the institutional protocol, BME is the most prominent in T2w images. Besides, in the most recent suggestion from the ASAS working group [11, 20], only the abovementioned T2w FS sequence is recommended. Images were retrieved from the Picture Archiving and Communication System in the Digital Imaging and Communications in Medicine format.

### Segmentation and labeling of ROIs

Two different heterogeneous regions of interest (CircleOriginal and FacetOriginal) were manually segmented and labeled using Research Portal V1.1 (United Imaging Intelligence, Co., Ltd., Shanghai, China). CircleOriginal and FacetOriginal were segmented and labeled back-to-back on oblique coronal MR images by two radiologists with 2 and 11 years of experience, respectively (Reader A and B). Prior to segmentation, both radiologists underwent training to ensure that the ROIs were drawn to the required standard. Boundaries of the ROIs were validated and redrawn by Reader B when necessary to reduce intra-reader bias and to better adhere to the segmentation criteria.

Next, CircleOriginal was automatically expanded by 15 mm in all directions to generate Circle, which represents a pair of circular ROIs of a defined size located within the SIJ and encompasses as much of abnormal findings as possible. Circle was segmented and expanded using a method inspired by a previous study [21].

FacetOriginal was further automatically expanded by 10 mm to generate Facet, a ROI that spans six consecutive slices in the oblique coronal plane, including articular space and subarticular region to 1 cm. Criteria for segmentation and expansion of Facet were performed as done in a previous study [19].

**Fig. 3:**
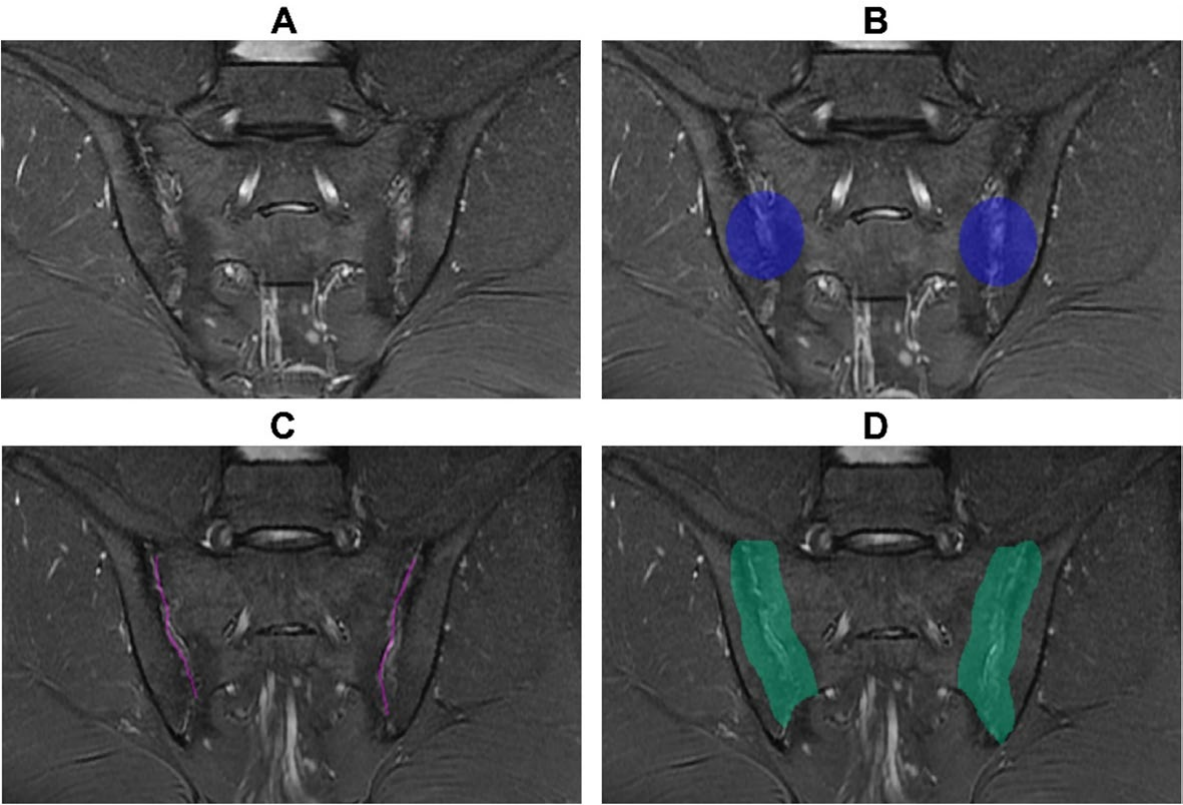
SIJ-MRI segmentation and expansion for four ROIs: CircleOriginal (**A**), Circle (**B**), Facet (**C**), and FacetOriginal (**D**). MR, magnetic resonance; SIJ, sacroiliac joint

All the expanded ROI boundaries were further validated and corrected by Reader B, to reduce possible bias and to better adhere to the segmentation criteria. An example of SIJ-MRI segmentation and expansion is shown in Fig. 3.

### Data preprocessing

The voxel value range of MRI images varies significantly across different machines and imaging modalities. To reduce the impact of voxel value outliers, we sorted all voxels’ values in each image and truncated them to the range of 0.5 to 99.5 percentile.

### Radiomics features

#### Feature extraction

We extracted corresponding radiomics features for each ROI in this study. Prior to extraction, various filters were applied to ROIs, including additive Gaussian white noise, Laplacian of Gaussian, shot noise, wavelet, binomial blur, recursive Gaussian, discrete Gaussian, curvature flow filters, and several simple filters, such as BoxMean and Normalize. All features were extracted using the PyRadiomics package (version 3.0.1) [25], and most of them adhered to the feature definitions provided by the Imaging Biomarker Standardization Initiative [26].

The handcrafted features were classified as (I) geometry, (II) intensity, and (III) texture. Geometry features capture the three-dimensional shape of the tumor. Intensity features represent the first-order statistical distribution of voxel intensities within the tumor. Texture features describe patterns or the second- and higher-order spatial distributions of intensities. Various methods were used to extract texture features, including the gray-level co-occurrence matrix, the gray-level run length matrix, the gray-level size zone matrix, and the neighborhood gray-tone difference matrix.

To evaluate the performance of the multi-ROI model, we fused the features extracted from each ROI to obtain fused features. We compared the performance of modeling using single-ROI features and fused features in our experiments.

#### Feature selection

##### Intraclass correlation coefficient (ICC)

To assess the robustness of image features against segmentation uncertainties, test–retest and inter-rater analyses were conducted. Test–retest analysis involving two segmentations was performed by one rater for each of the randomly selected 32 patients, while inter-rater analysis involving independent segmentations of ROIs was performed by two raters for another set of 32 randomly chosen patients. In the test–retest analysis, overall ICC was high for both Reader A (an average of 0.965 and 0.942 for Circle- and Facet-based features) and Reader B (an average of 0.963 and 0.956 for Circle- and Facet-based features). For inter-rater analysis, ICC was also high, averaging 0.965 and 0.944 for Circle- and Facet-based features respectively. The ICC was used to evaluate the features extracted from the multiple-segmented subregions. Features with an ICC ≥ 0.85 were considered robust and unaffected by segmentation uncertainties.

##### Feature pre-fusion

In this study, the performance of features derived from two different MRI ROIs, Circle and Facet, were compared. To assess whether features chosen from multiple ROIs outperformed those from a single ROI, prior further feature screening features extracted from the two ROIs were combined to obtain a fusion feature set. The remaining processes were the same as those for features extracted from one ROI and followed the same parameter configuration.

##### Standardization screening

Following the initial screening using ICC, all features were standardized using the *Z*-score method to ensure a normal distribution. Subsequently, *p*-values were calculated for all imaging features using the* t*-test. Only radiomic features with a* p*-value < 0.05 were retained for further analysis.

##### Correlation screening

Highly repeatable features were further analyzed using Pearson’s correlation coefficient to identify highly correlated features. To avoid redundancy, only one feature was retained when the correlation coefficient between any two features exceeded 0.9. To maximize feature representation while minimizing redundancy, a greedy recursive deletion strategy was used to remove features with the highest redundancy in the current set at each iteration. Additionally, the minimum Redundancy Maximum Relevance algorithm was used to further reduce feature redundancy. For each ROI, only the 64 most important features were retained.

##### Least absolute shrinkage and selection operator (LASSO) screening

The final features used to construct the radiomics signature (Rad_Sig) were selected using the LASSO regression model. LASSO shrinks regression coefficients, setting many irrelevant features’ coefficients to zero based on the regularization weight lambda (*λ*). The optimal *λ* value was determined using tenfold cross-validation with the minimum criterion, and *λ* with the lowest mean standard error was selected.

### Construction of the assessment model

After feature screening using LASSO, various machine learning models, including Logistic Regression, Support Vector Machine, Random Forest, ExtraTrees, and eXtreme Gradient Booting (XGBoost), were used to construct the assessment model.

In the training set, we performed a fivefold cross-validation and utilized the Grid Search algorithm for hyperparameter optimization. The best model parameters were selected based on their performance in the test set. Finally, with fixed hyperparameters, we trained the model using the entire training set (80%) and evaluated its performance in the remaining data set.

### Statistical analysis

We used Statistical Package for the Social Sciences 24.0 (IBM SPSS Inc., USA) for statistical analysis. Kolmogorov–Smirnov method was used to test the normality of the measurement data. Quantitative data were expressed as mean ± standard deviation (x̄ ± s). Chi-square test was used for comparison between the two groups. Student *t*-test was used for quantitative data. *p* < 0.05 indicated a statistically significant difference. The receiver operating characteristic (ROC) curve was used to analyze the performance of each model in the training set and test set. The efficiency of each model is evaluated by accuracy, the area under the curve (AUC), and its 95% confidence interval (CI), sensitivity, specificity, F1 score, true positive rate (TPR), false positive rate (FPR), and Youden index. Calibration curves were generated to evaluate the calibration performance of the model, and the clinical utility of the model was determined by decision curve analysis (DCA).

## Results

### Construction and evaluation of radiomics signature

We extracted 2286 handcrafted radiomic features of each model. The radiomics feature included first-order features, 14 different shape features, and other texture features. Additional file 1 provides details for the handcrafted features.

**Fig. 4:**
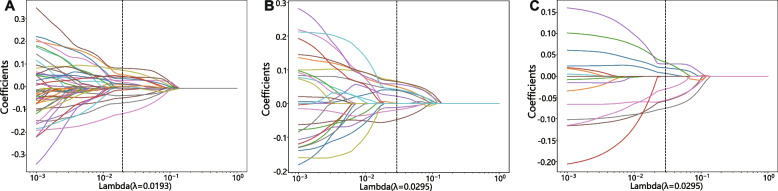
Coefficients of tenfold cross-validation; from left to right, the fusion feature set (left), features extracted from Circle (center), and features extracted from Facet (right)

**Fig. 5:**
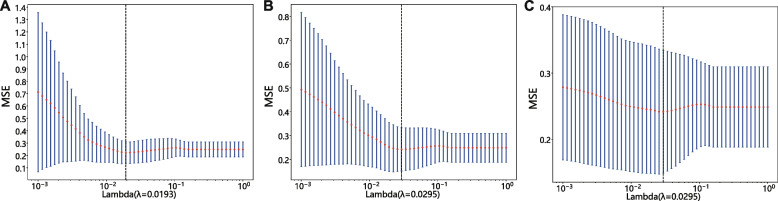
Mean standard error of tenfold cross-validation for the fusion feature set (left), features extracted from Circle (center), and features extracted from Facet (right), respectively

Seven Facet-derived features, 11 Circle-derived features and 18 features from the fusion feature set of nonzero LASSO coefficients were selected to constitute the Rad_Sig. These specific radiomics features are presented in Additional file 1. Their coefficients and the mean standard error of tenfold validation is shown in Figs. 4 and 5.

### Comparison of different models

**Fig. 6:**
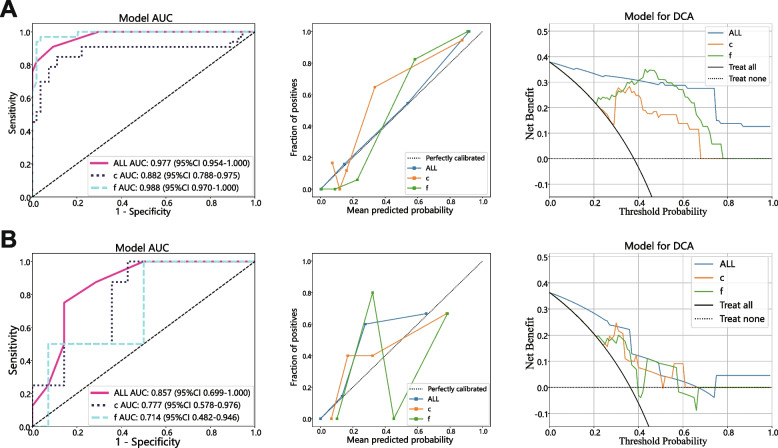
AUC-ROC (left), calibration (center), and decision (right) curves of different Rad_Sig in training (up) and testing (down) cohorts. The model based on fusion-set derived Rad_Sig and constructed with Random Forest outperforms the other two models. AUC, area under the curve; DCA, decision curve analysis; ROC, receiver operating characteristic; ROI, region of interest

For Rad_Sig comprised of Facet-derived features, Circle-derived features, and features from the fusion set, the best-performing models were constructed with XGBoost, ExtraTrees, and Random Forest respectively. The predictive capabilities and other statistical analysis results of these models are presented in Table 2. The AUC-ROC, calibration, and DCA curves of these models are shown in Fig. 6. Detailed comparisons between models constructed with various Rad_Sig and machine learning models are presented in the Additional file 1.

**Table 2:** axSpA activity prediction performance of models based upon different Rad_Sig

Models	Accuracy	AUC	95% CI	Sensitivity	Specificity	PPV	NPV	Youden	Cohort
Fusion	0.908	0.977	0.9536–0.9998	0.909	0.907	0.857	0.942	0.500	Train
Circle	0.874	0.882	0.7880–0.9752	0.848	0.889	0.824	0.906	0.362	Train
Facet	0.966	0.988	0.9703–1.0000	0.970	0.963	0.941	0.981	0.438	Train
**Fusion**	**0.818**	**0.857**	**0.6994**–**1.0000**	**0.750**	**0.857**	**0.750**	**0.857**	**0.361**	**Test**
Circle	0.727	0.777	0.5781–0.9755	1.000	0.571	0.571	1.000	0.300	Test
Facet	0.682	0.714	0.4822–0.9463	1.000	0.538	0.533	1.000	0.345	Test

*AUC* area under the curve, *CI* confidence interval, *PPV* positive predictive value, *NPV* negative predictive value

For the three machine learning models used to construct the best-performing models, their specific parameters are as follows:

- Random Forest: This model is configured with 4 estimators, a maximum depth of 6. The minimum number of samples required to split nodes is set to 2.
- XGBoost: This model is configured with 3 estimators. The maximum depth is set to 4.
- ExtraTrees: This model is configured with 8 estimators, a maximum depth of 4. The minimum number of samples required to split nodes is set to 2.

As indicated by bold type in Table 2, the model based on fusion-set derived Rad_Sig and constructed with Random Forest exhibited the best prediction performance in the test set while maintaining low overfitting. As shown in Fig. 6, it achieved a high AUC value; the calibration curve indicates that the calibration performance of the fusion-set derived model is better than the two other models, and the decision curve suggests that the fusion-set derived model has a better prediction performance. The results demonstrate that the Rad_Sig derived from the fusion feature set, which combines features from both ROIs, has a higher clinical application value.

## Discussion

In this research, we introduced a multi-ROI fusion radiomics model to predict axSpA disease activity using data for 109 patients. The results suggest MRI-based radiomics of the SIJ can distinguish axSpA activity, which could ultimately improve the therapeutic result and patient prognosis. To our knowledge, this is the only study in the literature that used a radiomics approach to determine axSpA activity.

Accurately judging axSpA disease activity is challenging, as the evaluation involves combining clinical, laboratory, and radiologic aspects of the patient. However, MRI is the most objective method to evaluate disease activity, especially inflammation presenting in the form of BME. Further, an abundant reserve of high-dimensional data can be extracted from MRIs using radiomics. In oncology, radiomics is well-developed to extract a vast array of quantitative characteristics from MRI data, allowing for the prediction of clinical outcomes [14].

Until now, four researchers have explored the application of radiomics in axSpA. In a cohort of 47 SpA patients, Tenorio et al. associated quantitative and radiomic biomarkers extracted from spectral attenuated inversion recovery (SPAIR) and short tau inversion recovery (STIR) sequences of SIJ MRI with various clinical indices to potentially compose a radiomic model for assessing SpA [20, 27]. Kepp et al. found that texture analysis is superior to qualitative assessment for differentiating sacroiliitis and degenerative changes using radiomics based on data of 90 patients [21]. Ye et al. used multivariable logistic regression to build a radiomics model using data of 638 patients (424 with axSpA and 214 non-axSpA) that showed performance in training and testing cohorts, with accuracy (0.78 vs. 0.74), sensitivity (0.75 vs. 0.71), and specificity (0.83 vs. 0.81). However, the clinical-radiomics nomogram model that incorporated radiomics with independent factors showed significant improvement in accuracy (0.82 vs.0.78), sensitivity (0.82 vs. 0.94), and specificity (0.83 vs. 0.62) in training and testing cohorts [19]. These studies included fewer cases or were focused on diagnosis rather than disease activity.

Many existing axSpA disease activity scales are based on SIJ-MRIs, including the Berlin Method [28] and the Spondyloarthritis Research Consortium of Canada (SPARCC) Scoring System [29]. Both scales require a trained professional to count the number of subchondral articular sections with BME. Therefore, using these scales is time-consuming and heavily depends on reader’s experience, which may have subjective problems that limit clinical application [30]. Moreover, patients with lower back pain or even healthy individuals may also be identified as having BME of SIJs, thus leading to low specificity of diagnosis by MRI alone [31–33]. Therefore, scale based on imagery oftentimes overdiagnoses axSpA activity. These scoring systems do not measure subchondral bone erosions or fat deposition, both of which could be caused by and potentially measure axSpA activity. Zheng et al. explored the possibility of replacing these scoring systems with a radiomics-based model. Researchers found that the radiomics model can distinguish between high and low SPARCC groups, and the resulting radiomics model is significantly correlated to the SPARCC score [34].

In the present study, a combined ROI model accurately discriminated between active and non-active axSpA, with high accuracy (0.90 vs. 0.81), sensitivity (0.90 vs. 0.75), and specificity (0.90 vs. 0.85) in the training and testing cohorts. This suggests that SIJ-MRI-based radiomics biomarkers and models can discriminate axSpA activity, which is a drastic improvement from the results of previous studies that used SIJ-MRI-based scales to assess activity [3, 35]. By integrating relevant clinical factors into our model, such as age, gender, patient-reported outcome measures, and laboratory results, correlations between radiomics biomarkers and these clinical factors can be further explored, and the efficiency and accuracy of our model may be further improved.

In the present study, while models derived from Facet-based features outperformed those from Circle in the train set, Circle-based model outperformed the Facet-based model in the test set. Circle represents what the reader considers to be the section of SIJ that is most dominated by inflammatory lesions, mostly BME. BME is considered as the MRI finding indicative of inflammation and axSpA activity. However, axSpA is accompanied by other chronic structural changes, including erosion, osteosclerosis, fatty infiltration, and ankylosis. Facet is a larger and less arbitrary portion of the SIJ, that includes more of the articular and subarticular regions. Thus, findings other than BME relevant to axSpA disease activity are included within. The fusion-ROI model combines the best of both worlds and outperforms both Circle- and Facet-based models in the train set and the test set.

There are some limitations to our study. First, this is a retrospective study and might have selection bias; prospective studies are required to confirm our findings. Second, our results were derived from data of a single institution, and to enhance reproducibility, external validation will be required. Third, two similar machines with the same scanning protocol were used. While this may provide better comparability across the MR images, a radiomics model based on images selected from various MR scanners may be more robust and better suited for a clinical setting. Fourth, manual ROI segmentation is complex and time-consuming. Automatic ROI segmentation techniques are required to improve its reliability and reproducibility. Moreover, only one sequence (T2w FS FSE) was chosen to extract images. Other studies included more sequences sensitive to inflammation, such as SPAIR and STIR. Sequences more sensitive to structural changes, especially T1-weighted spin echo without FS, were not included in our protocol. With more imaging modalities, more radiomics features can be extracted to potentially construct a more robust and sensitive model.

## Conclusion

In summary, we successfully trained a multi-ROI fusion radiomics model to predict axSpA activity. Our findings indicate that the combined model has the best discriminatory ability in determining MRI of SIJ between active and non-active axSpA patients. Further research is needed to explore the potential of radiomics in the field of axSpA.

### Supplementary Information

**Additional file 1.** Hand-crafted feature extraction and detailed comparisons between models constructed with various Rad_Sig and machine learning models.

## Data Availability

The data underlying this article cannot be shared publicly for the protection of the privacy of individuals who participated in the study. The data may be shared upon reasonable request to the corresponding author.

## Abbreviations

ASDAS: Ankylosing Spondylitis Disease Activity Score
ASAS: Assessment of SpondyloArthritis International Society
axSpA: Axial spondyloarthritis
BASDAI: Bath Ankylosing Spondylitis Disease Activity Index
BME: Bone marrow edema
CRP: C-reactive protein
DCA: Decision curve analysis
FSE: Fast spin-echo
FS: Fat suppressed
ICC: Intraclass correlation coefficient
LASSO: Least absolute shrinkage and selection operator
MRI: Magnetic resonance imaging
Rad_Sig: Radiomics signature
ROI: Region of interest
SIJ: Sacroiliac joint
SPAIR: Spectral attenuated inversion recovery
SPARCC: Spondyloarthritis Research Consortium of Canada
STIR: Short tau inversion recovery
T2w: T2 weighted
