# Supplementary material for: Assessment of axial spondyloarthritis activity using a magnetic resonance imaging-based multi-region-of-interest fusion model
Source: Arthritis Res Ther. 2023 Nov 24;25:227. doi: 10.1186/s13075-023-03193-6 (PMC10668377; doi:10.1186/s13075-023-03193-6)

**Supplementary materials:**

**Hand-crafted feature extraction**

In this study we employed a feature-based approach to extract and quantify meaningful and reliable information from images. The hand-crafted radiomic feature extraction algorithms were standardized as done by referring to the Image Biomarker Standardisation Initiative (IBSI) The features could be divided into three groups: shape features, global texture features and local texture features. Shape features and global texture features were the most common means for evaluating the outline and the textual parameters of regions and have been verified as useful tools to differentiate patients based on histologic type, progress, and prognosis. Local texture features seem like an extension of the region intensity measurement in the conventional radiological analysis pipeline and measure more properties of local intensity histogram distribution. The process is described in detail below.

Shape features describe the morphological property of the segmentation. 2D shape features were generated from the image without filtration ($X$).

Global texture features calculate the pattern of pixel-intensity distribution in the ROI. Global texture features including the features from the categories of first-order texture (i.e., histogram [H] feature), second-order texture (i.e., GLCM feature) and high-order texture (i.e., GLRLM feature) were computed from the image without/after filtration (${X,X}_{LL},X_{HH}$). Note that the image intensity was discretized to a fixed number of 25 bins to reduce the influence from image noise.

Local texture features estimate the statistical characteristics of texture value computed from the neighborhood of each pixel. In this study, as reported, local texture features measured multi-percentiles of distribution of histogram-/GLCM-feature value computed per pixel by using a 5 × 5 patch centered at each pixel. It is computed from the image without/after filtration (${X,X}_{LL},X_{HH}$).

**Fusion Set:**

**Features Selected**:


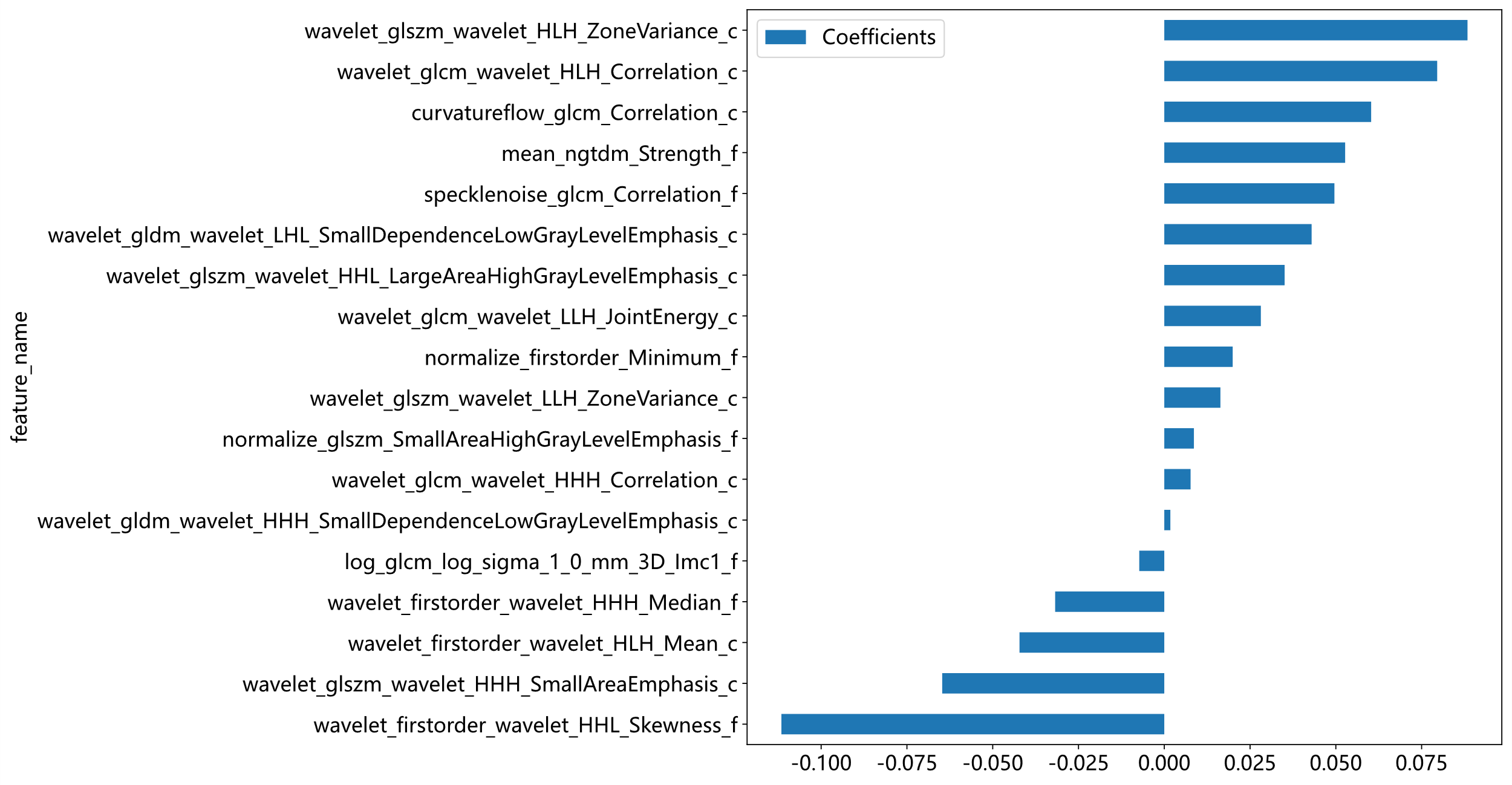


**Prediction performance of models**:

| Model | Accuracy | AUC | 95% CI | Sensitivity | Specificity | PPV | NPV | Precision | Recall | F1 | Threshold | Task |
| --- | --- | --- | --- | --- | --- | --- | --- | --- | --- | --- | --- | --- |
| LR | 0.885 | 0.893 | 0.8072 - 0.9785 | 0.818 | 0.926 | 0.871 | 0.893 | 0.871 | 0.818 | 0.844 | 0.473 | Train |
| LR | 0.682 | 0.696 | 0.4657 - 0.9272 | 0.875 | 0.571 | 0.538 | 0.889 | 0.538 | 0.875 | 0.667 | 0.239 | Test |
| SVM | 0.954 | 0.955 | 0.8999 - 1.0000 | 0.909 | 0.981 | 0.968 | 0.946 | 0.968 | 0.909 | 0.937 | 0.362 | Train |
| SVM | 0.636 | 0.661 | 0.4116 - 0.9099 | 1.000 | 0.429 | 0.500 | 1.000 | 0.500 | 1.000 | 0.667 | 0.225 | Test |
| RandomForest | 0.908 | 0.977 | 0.9536 - 0.9998 | 0.909 | 0.907 | 0.857 | 0.942 | 0.857 | 0.909 | 0.882 | 0.500 | Train |
| RandomForest | 0.818 | 0.857 | 0.6994 - 1.0000 | 0.750 | 0.857 | 0.750 | 0.857 | 0.750 | 0.750 | 0.750 | 0.361 | Test |
| ExtraTrees | 0.931 | 0.934 | 0.8660 - 1.0000 | 0.879 | 0.963 | 0.935 | 0.929 | 0.935 | 0.879 | 0.906 | 0.390 | Train |
| ExtraTrees | 0.818 | 0.777 | 0.5447 - 1.0000 | 0.625 | 0.929 | 0.833 | 0.812 | 0.833 | 0.625 | 0.714 | 0.413 | Test |
| XGBoost | 0.954 | 0.991 | 0.9793 - 1.0000 | 0.970 | 0.944 | 0.914 | 0.981 | 0.914 | 0.970 | 0.941 | 0.431 | Train |
| XGBoost | 0.727 | 0.647 | 0.3729 - 0.9218 | 0.500 | 0.923 | 0.667 | 0.750 | 0.667 | 0.500 | 0.571 | 0.488 | Test |


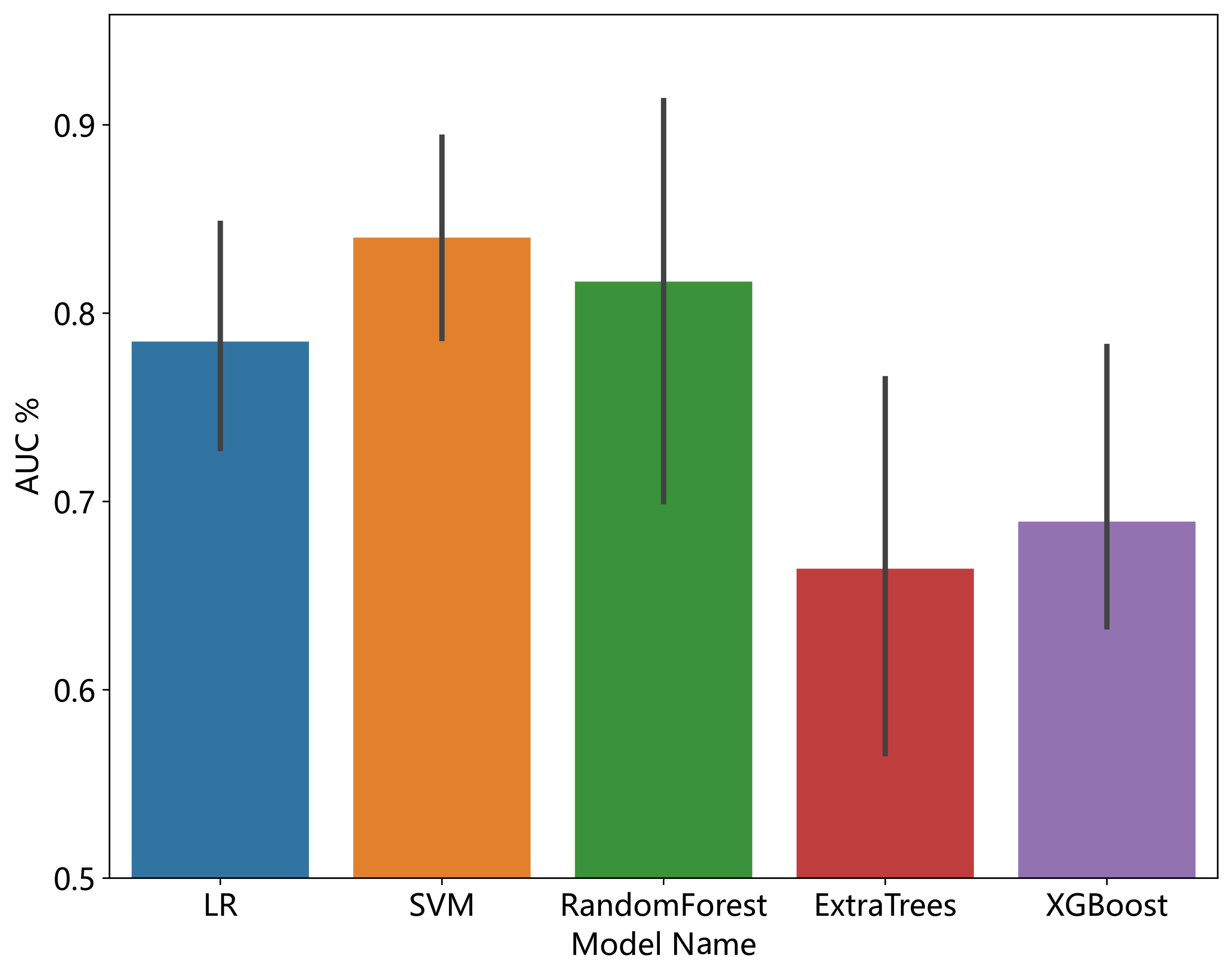


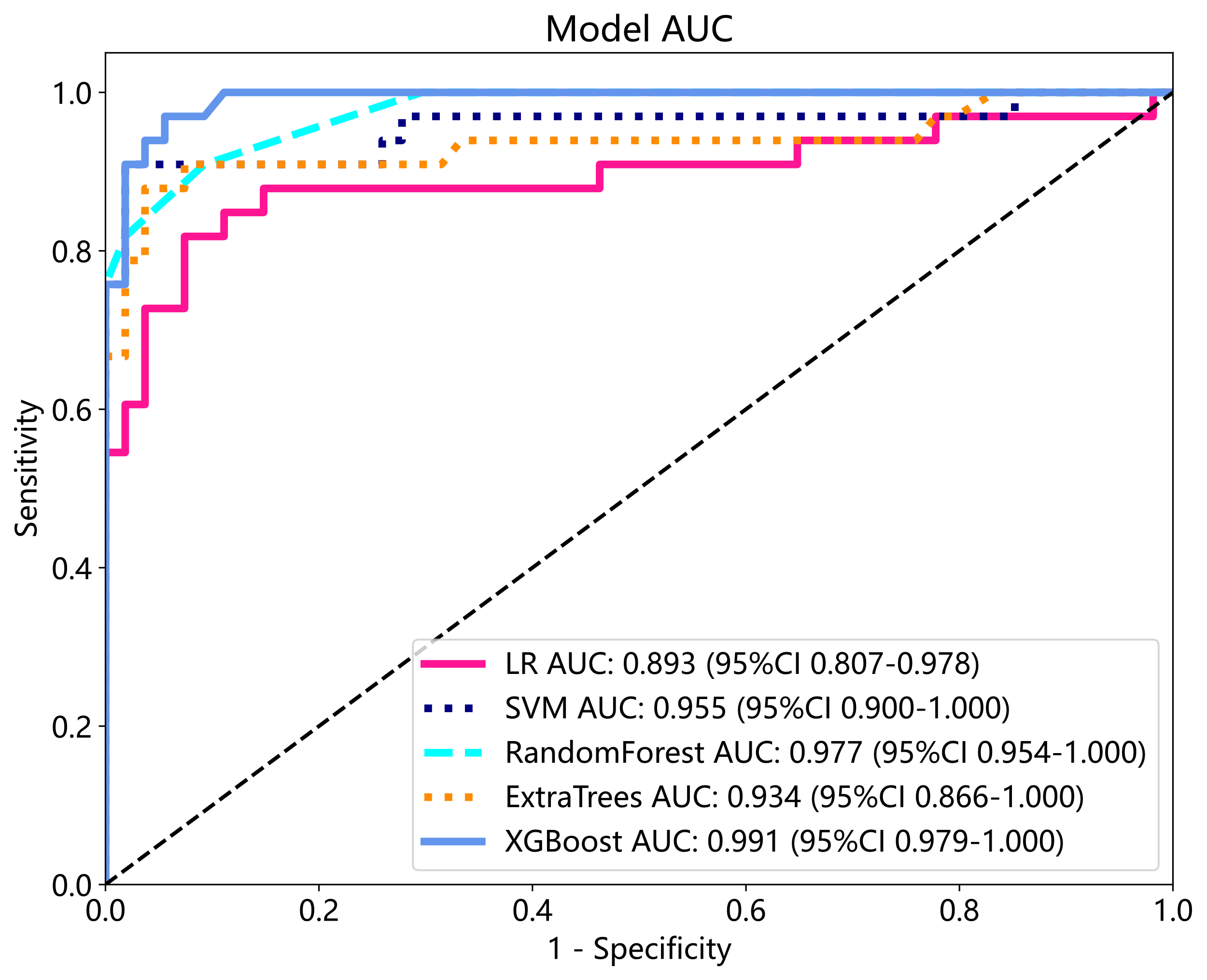


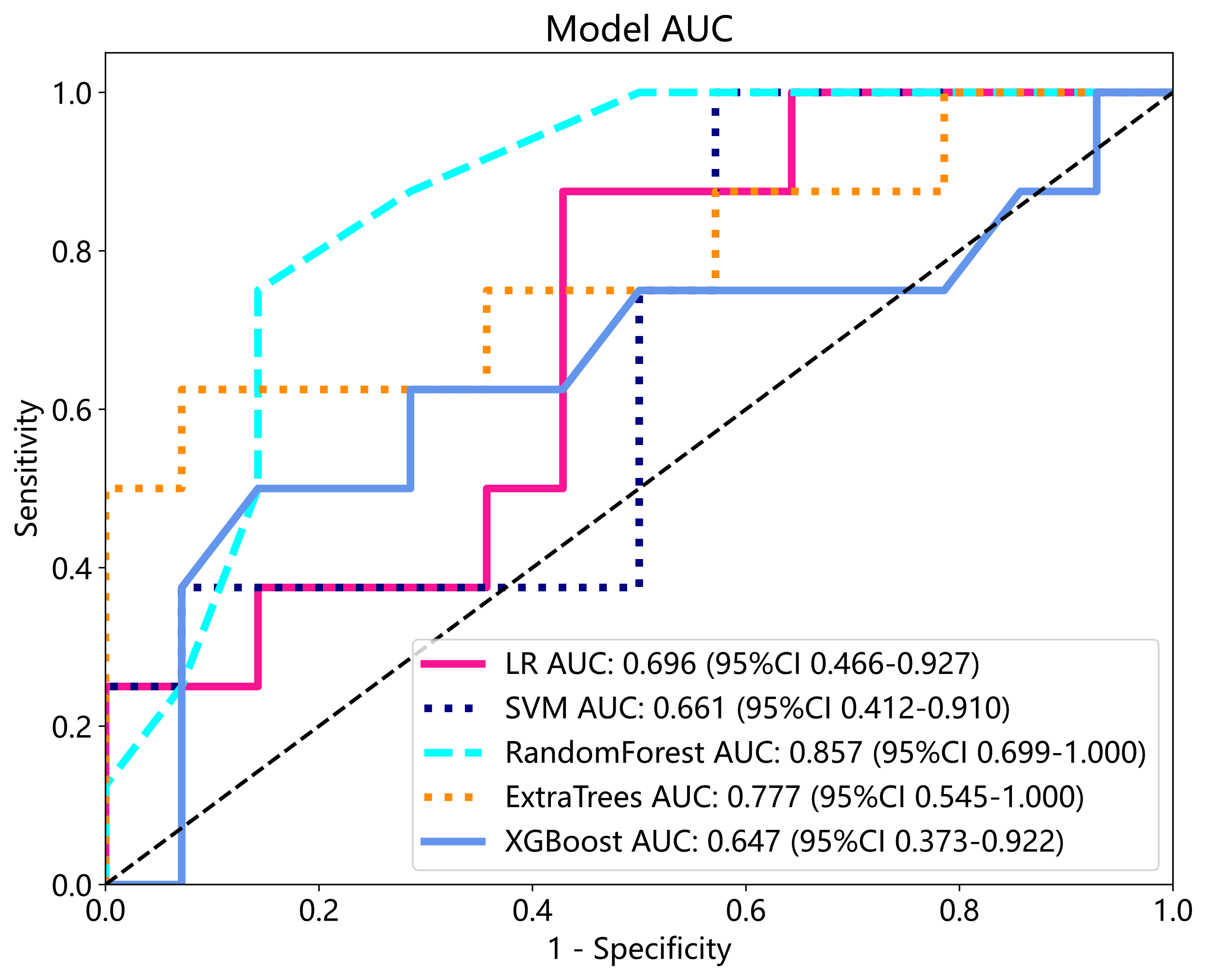


**Circle-derived features**

**Features Selected**:


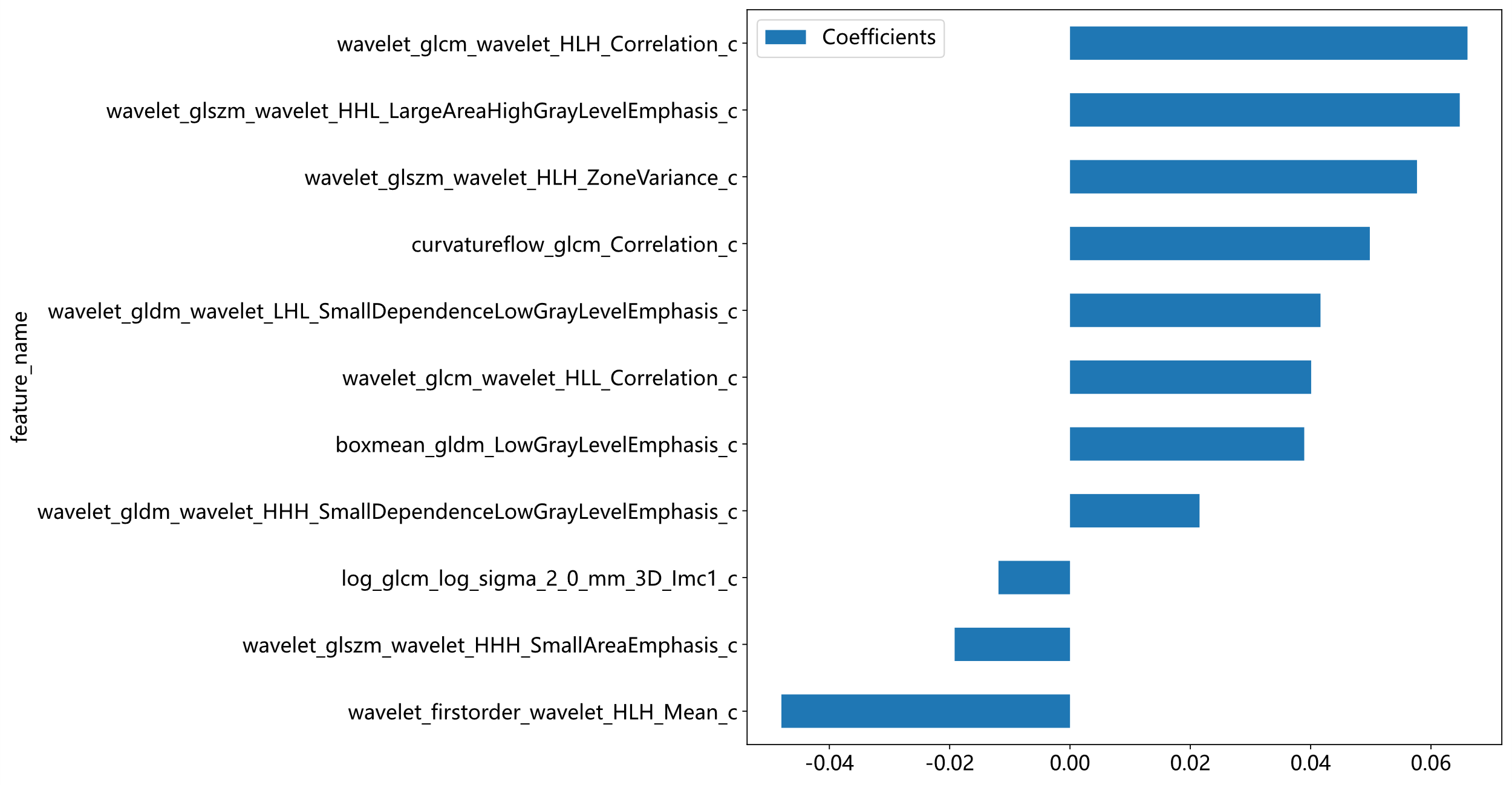


**Prediction performance of models**:

| Model | Accuracy | AUC | 95% CI | Sensitivity | Specificity | PPV | NPV | Youden | Task |
| --- | --- | --- | --- | --- | --- | --- | --- | --- | --- |
| LR | 0.782 | 0.823 | 0.7297 - 0.9167 | 0.788 | 0.778 | 0.684 | 0.857 | 0.375 | Train |
| LR | 0.682 | 0.705 | 0.4715 - 0.9392 | 0.750 | 0.643 | 0.545 | 0.818 | 0.229 | Test |
| SVM | 0.874 | 0.882 | 0.7880 - 0.9752 | 0.848 | 0.889 | 0.824 | 0.906 | 0.362 | Train |
| SVM | 0.727 | 0.777 | 0.5781 - 0.9755 | 1.000 | 0.571 | 0.571 | 1.000 | 0.300 | Test |
| RandomForest | 0.943 | 0.985 | 0.9676 - 1.0000 | 0.970 | 0.926 | 0.889 | 0.980 | 0.470 | Train |
| RandomForest | 0.727 | 0.714 | 0.4654 - 0.9632 | 0.875 | 0.692 | 0.583 | 0.900 | 0.333 | Test |
| ExtraTrees | 0.874 | 0.910 | 0.8451 - 0.9747 | 0.697 | 0.981 | 0.958 | 0.841 | 0.421 | Train |
| ExtraTrees | 0.773 | 0.607 | 0.3040 - 0.9103 | 0.375 | 1.000 | 1.000 | 0.737 | 0.561 | Test |
| XGBoost | 0.920 | 0.980 | 0.9576 - 1.0000 | 0.939 | 0.907 | 0.861 | 0.961 | 0.418 | Train |
| XGBoost | 0.773 | 0.670 | 0.4060 - 0.9333 | 0.500 | 0.929 | 0.800 | 0.765 | 0.508 | Test |


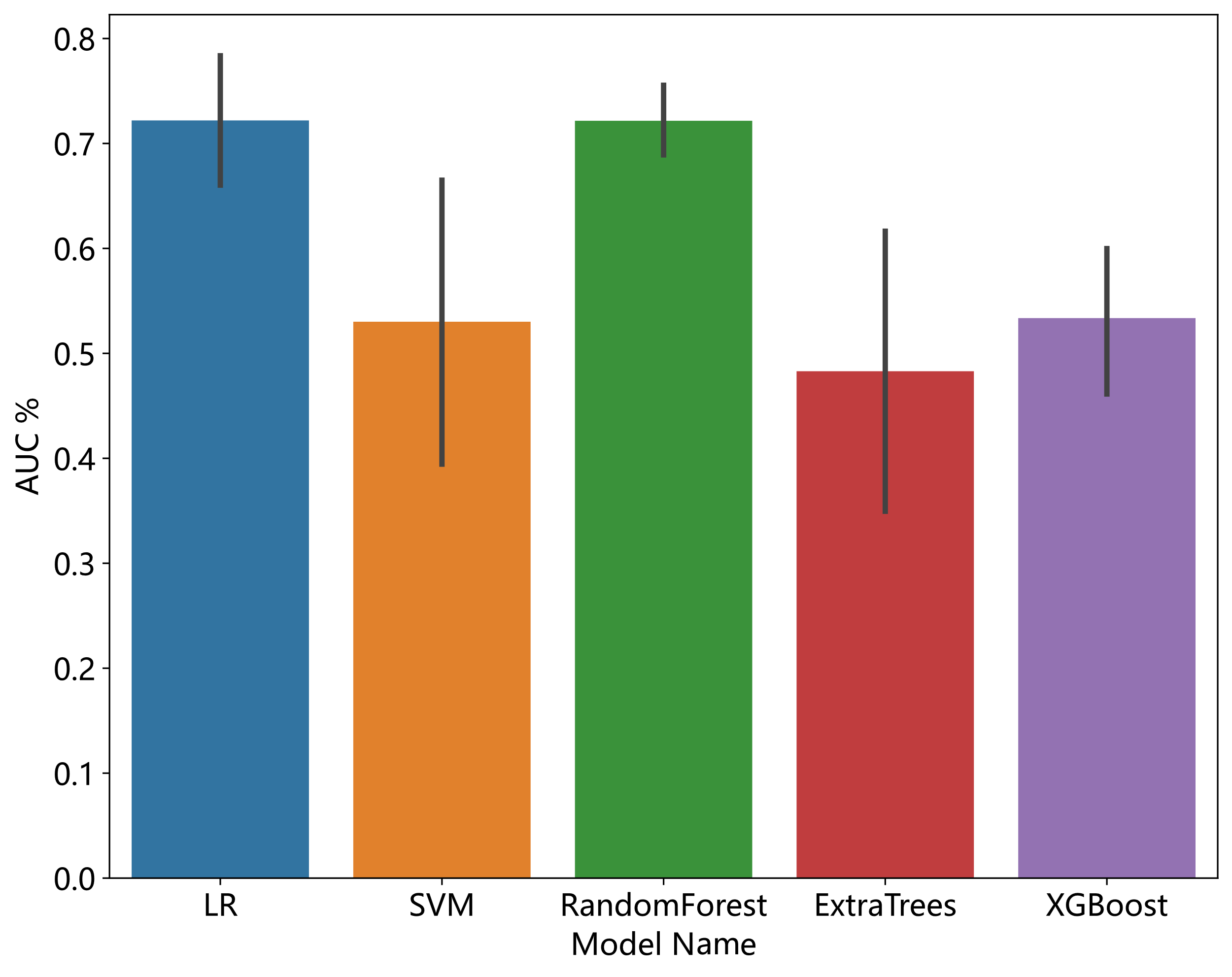


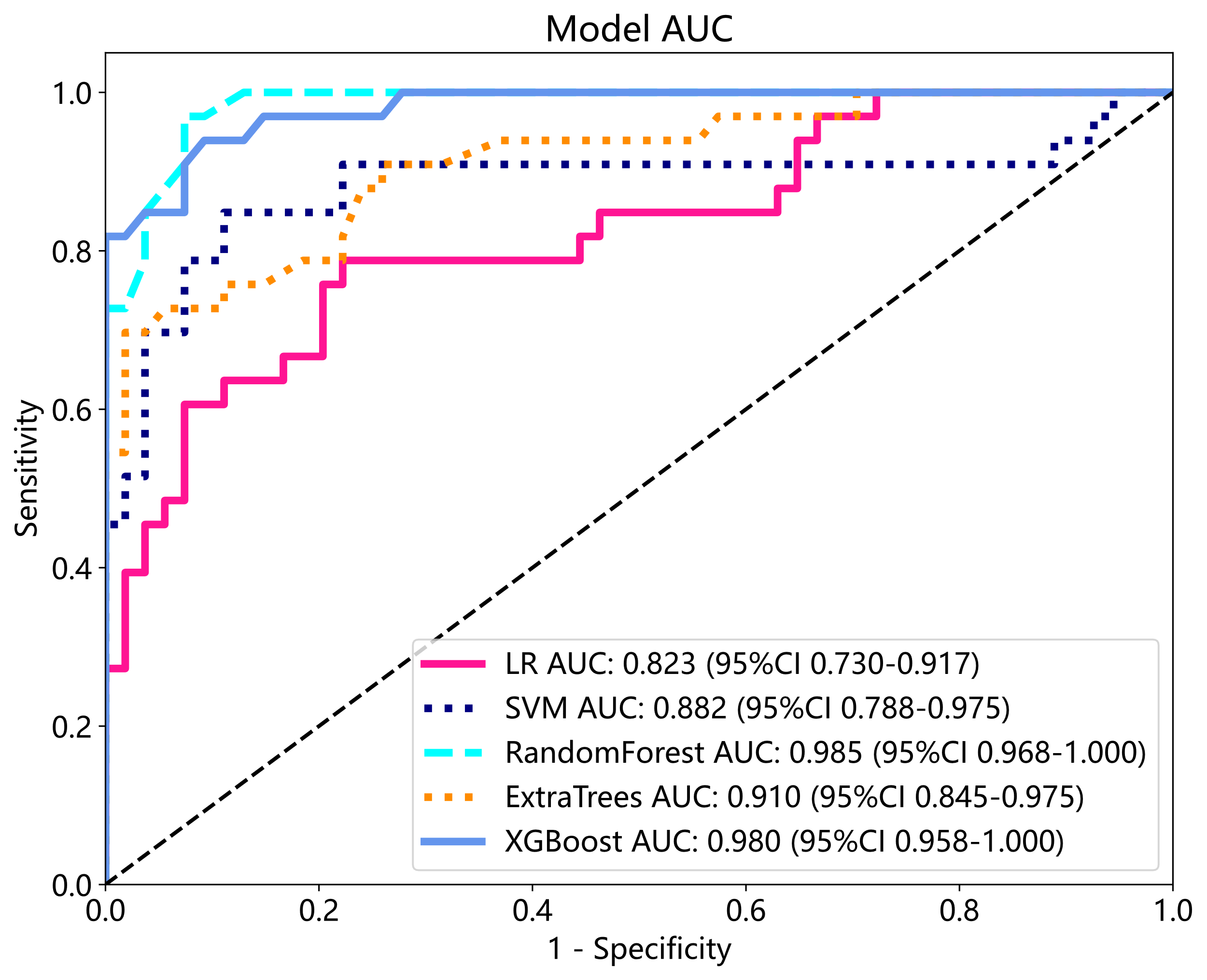


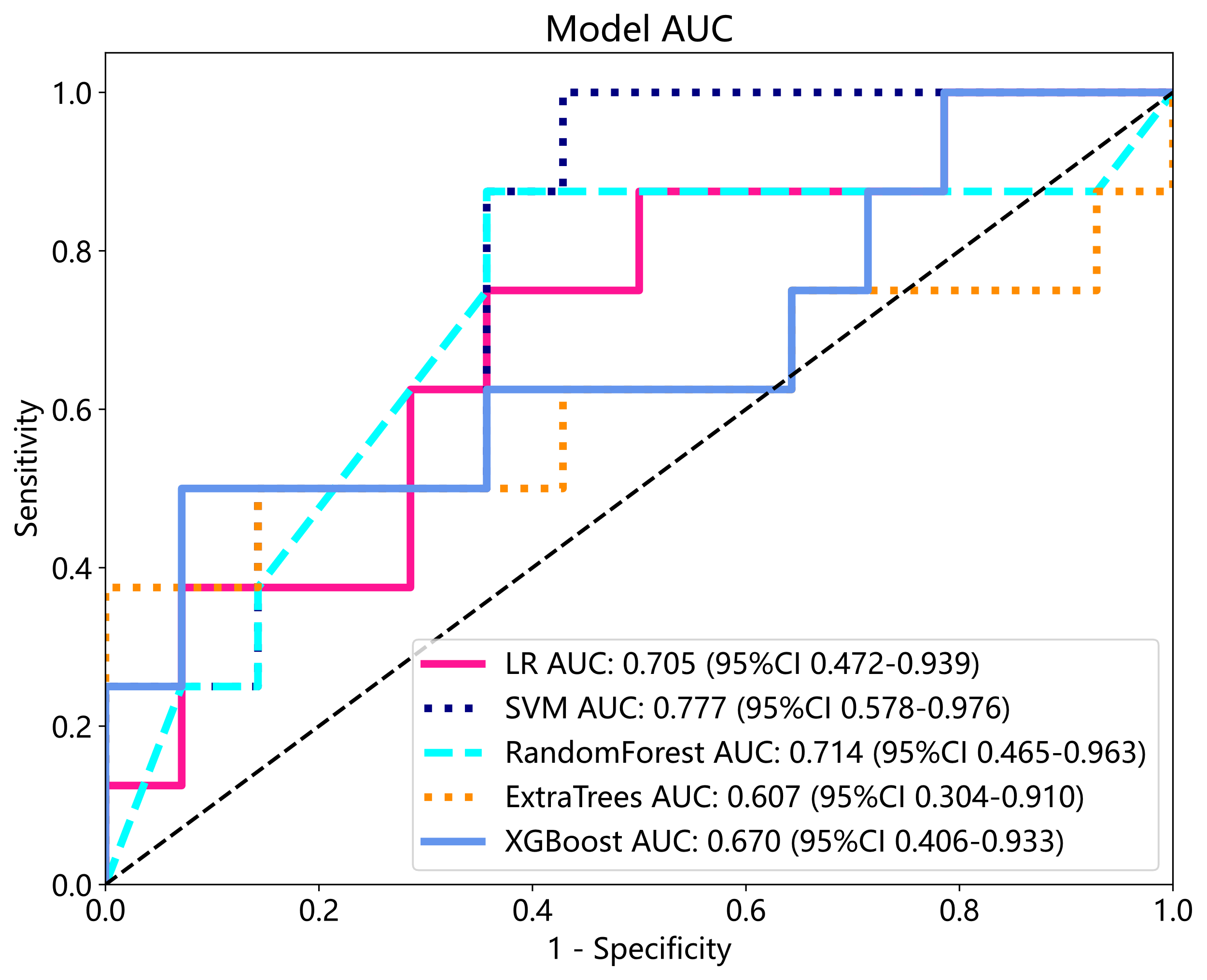


**Facet-derived features**

**Features Selected**:


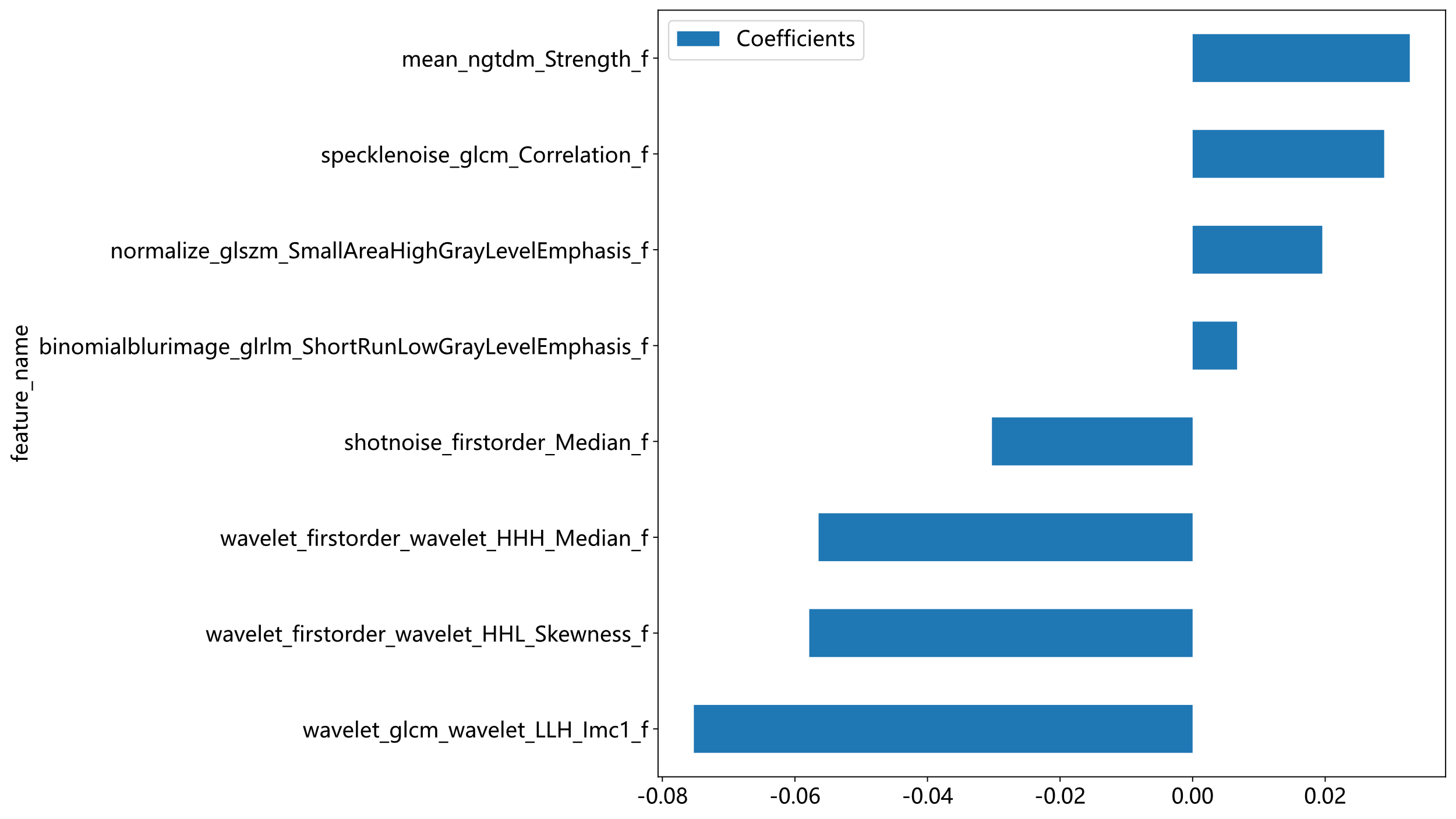


**Prediction performance of models**:

| Model | Accuracy | AUC | 95% CI | Sensitivity | Specificity | PPV | NPV | Youden | Task |
| --- | --- | --- | --- | --- | --- | --- | --- | --- | --- |
| LR | 0.747 | 0.756 | 0.6487 - 0.8642 | 0.606 | 0.833 | 0.690 | 0.776 | 0.430 | Train |
| LR | 0.545 | 0.545 | 0.2870 - 0.8022 | 0.875 | 0.385 | 0.437 | 0.833 | 0.259 | Test |
| SVM | 0.816 | 0.924 | 0.8730 - 0.9755 | 0.970 | 0.722 | 0.681 | 0.975 | 0.310 | Train |
| SVM | 0.545 | 0.589 | 0.3410 - 0.8375 | 1.000 | 0.308 | 0.444 | 1.000 | 0.290 | Test |
| RandomForest | 0.931 | 0.984 | 0.9660 - 1.0000 | 0.970 | 0.907 | 0.865 | 0.980 | 0.453 | Train |
| RandomForest | 0.591 | 0.598 | 0.3559 - 0.8405 | 0.875 | 0.500 | 0.467 | 0.857 | 0.250 | Test |
| ExtraTrees | 0.885 | 0.930 | 0.8796 - 0.9813 | 0.818 | 0.926 | 0.871 | 0.893 | 0.414 | Train |
| ExtraTrees | 0.500 | 0.500 | 0.2405 - 0.7595 | 1.000 | 0.231 | 0.421 | 1.000 | 0.278 | Test |
| XGBoost | 0.966 | 0.988 | 0.9703 - 1.0000 | 0.970 | 0.963 | 0.941 | 0.981 | 0.438 | Train |
| XGBoost | 0.682 | 0.714 | 0.4822 - 0.9463 | 1.000 | 0.538 | 0.533 | 1.000 | 0.345 | Test |


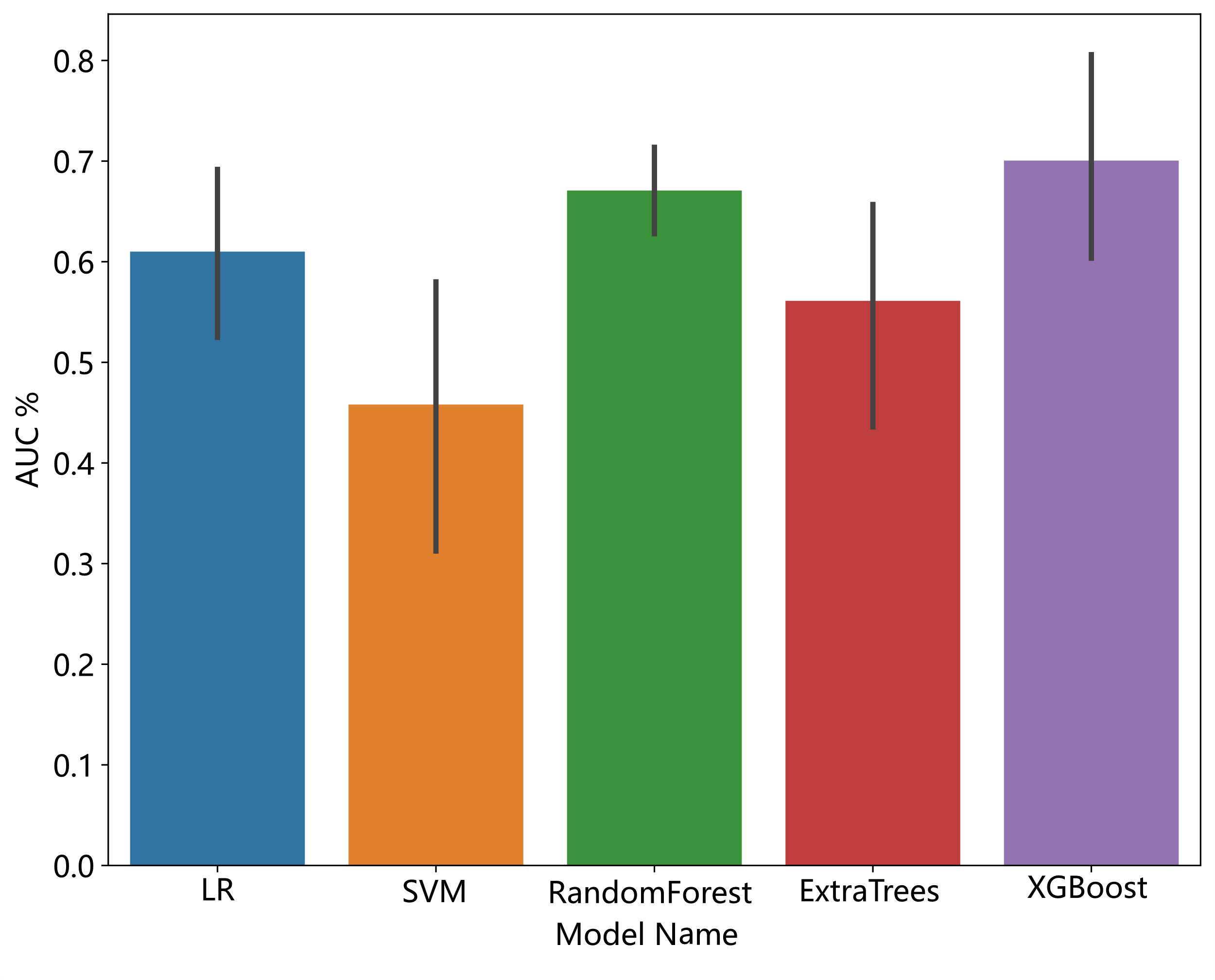


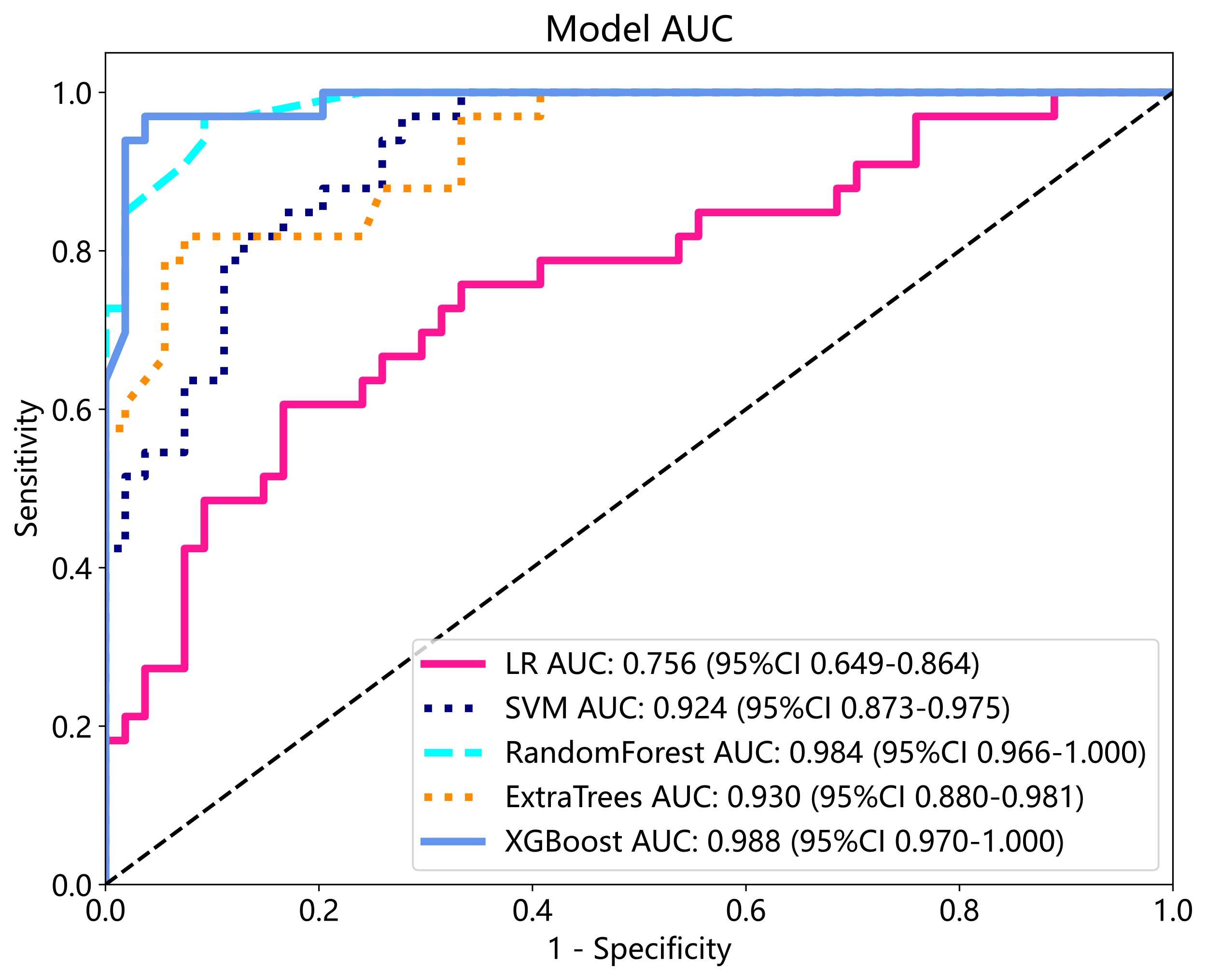


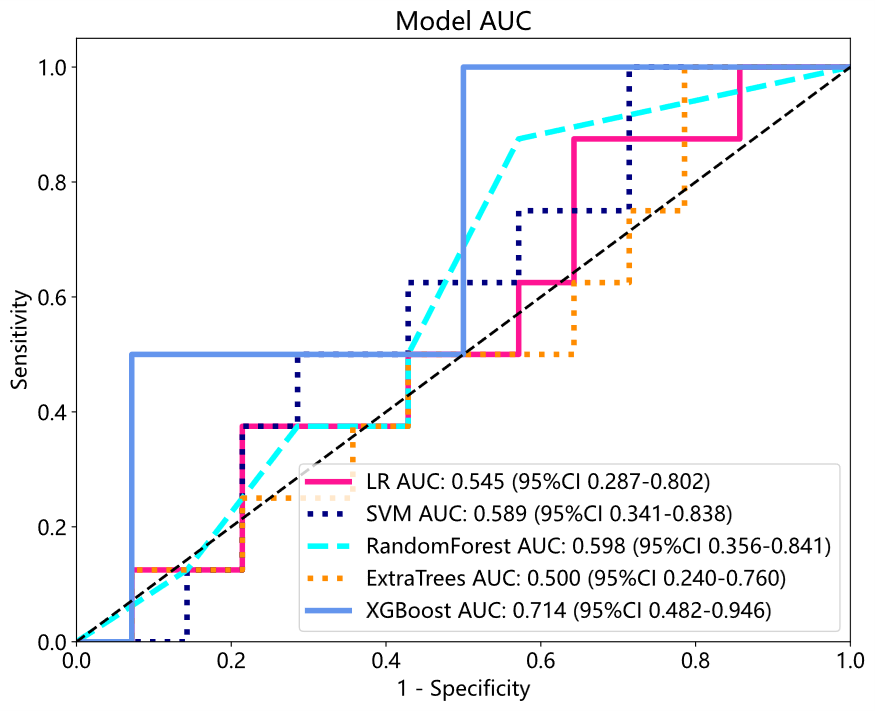

Supplement: Supplementary file 1 — Additional file 1. Hand-crafted feature extraction and detailed comparisons between models constructed with various Rad_Sig and machine learning models. [file 13075_2023_3193_MOESM1_ESM.docx]
